# Long-term effects of cognitive training in Parkinson’s disease: A randomized, controlled trial

**DOI:** 10.1016/j.prdoa.2023.100204

**Published:** 2023-06-07

**Authors:** Tim D. van Balkom, Odile A. van den Heuvel, Henk W. Berendse, Ysbrand D. van der Werf, Rob H. Hagen, Tanja Berk, Chris Vriend

**Affiliations:** a Amsterdam UMC, Vrije Universiteit Amsterdam, Psychiatry, De Boelelaan 1117, Amsterdam, The Netherlands; b Amsterdam UMC, Vrije Universiteit Amsterdam, Anatomy & Neurosciences, De Boelelaan 1117, Amsterdam, The Netherlands; c Amsterdam UMC, Vrije Universiteit Amsterdam, Neurology, De Boelelaan 1117, Amsterdam, The Netherlands; d Amsterdam Neuroscience, Neurodegeneration, Amsterdam, The Netherlands; e Amsterdam Neuroscience, Compulsivity, Impulsivity & Attention, Amsterdam, The Netherlands; f Dutch Parkinson’s Disease Association, PO Box 46, 3980 CA Bunnik, The Netherlands

**Keywords:** Parkinson’s disease, Cognitive training, RCT, Cognitive impairment, Long-term effects

## Abstract

**Background:**

Computerized cognitive training may be promising to improve cognitive impairment in Parkinson’s disease and has even been suggested to delay cognitive decline. However, evidence to date is limited. The aim of this study was to assess the durability of eight-week cognitive training effects at up to two years follow-up.

**Methods:**

One hundred and thirty-six (1 3 6) individuals with Parkinson’s disease, subjective cognitive complaints but without severe cognitive impairment (Montreal Cognitive Assessment ≥ 22) participated in this double-blind RCT. Participants underwent an eight-week home-based intervention of either adaptive, computerized cognitive training with *BrainGymmer* (n = 68) or an active control (n = 68). They underwent extensive neuropsychological assessment, psychiatric questionnaires and motor symptom assessment at baseline and one and two years after the intervention. We used mixed-model analyses to assess changes in cognitive function at follow-up and performed Fisher’s exact tests to assess conversion of cognitive status.

**Results:**

There were no group differences on any neuropsychological assessment outcome at one- and two-year follow-up. Groups were equally likely to show conversion of cognitive status at follow-up. A considerable amount of assessments was missed (1y: n = 27; 2y: n = 33), most notably due to COVID-19 regulations.

**Conclusions:**

Eight-week cognitive training did not affect long-term cognitive function in Parkinson’s disease. Future studies may focus on one cognitive subgroup to enhance reliability of study results. Intervention improvements are needed to work towards effective, lasting treatment options.

## Highlights

• Eight-week cognitive training did not improve cognitive function in the long term.
• No effects were measurable on delaying cognitive decline.
• The cognitive training group showed fewer depressive symptoms at follow-up, warranting further study.
• Booster sessions seem needed to retain cognitive training effects.
• Future studies are needed targeting specific stages of cognitive decline.

## 1 Background

In Parkinson’s disease (PD), the evidence from *meta*-analyses of small clinical trials suggests minor to modest positive effects of cognitive training on cognitive function [1]. Two studies have shown long-term (up to 18 months) cognitive stabilization or improvement after a 6- and 13-week cognitive training [2], [3], potentially due to cognitive training strengthening neural networks and thereby enhancing the resilience of the brain [4]. However, these studies were relatively small (n = 15–47) and *meta*-analyses have called for adequately blinded and controlled studies.

In the COGnitive Training In Parkinson Study (COGTIPS), we assessed the effects of eight-week computerized, multi-domain cognitive training compared with an active control condition in a sample of 136 individuals with PD. At post-intervention, cognitive training did not improve the primary outcome (executive function), but did induce improvements on processing speed and regional changes in structural and functional connectivity [5], [6], [7]. Here we report the effects of the intervention after one and two years of follow-up. We hypothesized cognitive training effects to endure at follow-up and to reduce conversion to mild cognitive impairment (PD-MCI) or dementia (PD-D).

## 2 Methods

### 2.1 Study design and participants

This study was part of the COGTIPS double-blind RCT to assess the superiority of eight-week computerized cognitive training over an active control condition in accordance with CONSORT guidelines (ClinicalTrials.gov: NCT02920632). A detailed overview of the trial methodology [8] and the primary and main secondary outcomes at post-intervention and at six-months follow-up [5], [6], [7] were reported previously.

We enrolled 140 individuals with PD at the Amsterdam University Medical Centers, location VUmc, with a) significant subjective cognitive complaints (PD Cognitive Functional Rating Scale score > 3), b) Montreal Cognitive Assessment (MoCA) score ≥ 22, c) Hoehn and Yahr stage < 4, d) Beck Depression Inventory (BDI) score ≤ 18, e) home access to a computer or tablet with internet connection and f) no indications of current drug- or alcohol abuse, impulse control disorders, psychotic symptoms, or history of traumatic brain injury. All participants gave written informed consent and the study was approved by the Medical Ethical Committee of VUmc.

### 2.2 Procedures

Eligible participants were randomized over a cognitive training condition and an active control condition in an 1:1 fashion, stratified by education level. Participants were not aware of the content of either condition, and both participants and assessors were blinded to the condition allocation.

All participants performed 24 sessions of a 45-minute online ‘gamified’ intervention for a duration of eight weeks on the *Braingymmer* platform (https://www.braingymmer.com/en). The cognitive training condition consisted of thirteen training games in each session that adapted to the individuals’ training capacity and focused on multiple cognitive functions, most notably executive function, but also attention, processing speed and working memory. The active control condition consisted of simple, non-adaptive games (solitaire, trivia questions, and hangman) that were sequentially performed for 15 min each.

The current report describes the trial results at one-year (T3) and two-year follow-up (T4) relative to baseline (T0). At each time-point participants underwent extensive neuropsychological assessment, questionnaires, interviews and motor assessment. See Supplementary Table S1 for an overview of all assessments. Dependent on governmental regulations due to COVID-19, we adapted assessment procedures by using plexiglass splash guards and face shields.

### 2.3 Outcomes

Outcomes in the current study were secondary endpoints of the COGTIPS clinical trial [8]. We assessed the long-term effects of cognitive training relative to the active control condition on executive function as primary outcome, as 1) executive function is most prevalently affected already early in PD [9], and 2) many studies to date have focused on the effects of cognitive training on executive function [1]. We measured executive function with accuracy (the primary outcome at T1) and response time on a computerized Tower of London task, that consisted of 100 pseudo-randomized trials with difficulty ranging from one-step to five-step solutions (task-load S1-S5). Other cognitive outcomes included the neuropsychological assessment instruments reported in Supplementary Table S1. Subjective cognitive complaints were measured with the PD Cognitive Functional Rating Scale and the Cognitive Failures Questionnaire.

We assessed group differences in conversion to PD-MCI or PD-D at T3 and T4 by classifying participants according to the most recent clinical diagnostic criteria [10], [11] to normal cognition, PD-MCI or PD-D, and defining conversion as ‘Cognitive worsening’, ‘Stable cognitive function’, or ‘Cognitive improvement’ at T3 and T4 relative to their T0 status.

Exploratory outcomes included psychiatric symptoms (see Supplementary Table S1). We assessed potential confounding factors including motor symptom severity (Unified PD Rating Scale part III), Hoehn & Yahr stage, levodopa equivalent daily dosage, and cognitive reserve (Cognitive Reserve Index questionnaire).

### 2.4 Statistical methods

Demographic and clinical variables at follow-up were compared between groups with analyses of variance, non-parametric Mann–Whitney U tests, or Fisher’s exact tests. We performed mixed-model analyses on the intention-to-treat population to assess the effect of cognitive training on cognitive function. We modelled condition and time (T1-T4) as independent variables, corrected for baseline performance, and additionally added age, sex and education level as covariates. We assessed group differences on the Tower of London task-load S1-S5 using multivariate mixed-models, where we used the *z*-transformed mean accuracy or response time on task-load S1-S5 as multivariate outcome, and added a random intercept at participant level to correct for correlation of the multivariate outcome within participants. Group differences on the neuropsychological outcomes and psychiatric symptom questionnaires were assessed using univariate mixed-model analyses, where we corrected for multiple comparisons while incorporating the mean correlation of the outcomes (Dubey/Armitage-Parmar method of Sidak’s adjustment; critical p-value neuropsychological tests: α = 0.008, critical p-value psychiatric questionnaires: α = 0.023).

We assessed the association between condition and conversion of cognitive status at T3 and T4 using Fisher’s exact tests, and estimated odds ratios of the ‘Cognitive worsening’ versus the ‘Stable cognitive function’ and ‘Cognitive improvement group’ in the per-protocol sample. We ran statistical analyses in SPSS version 26.0 (Armonk, NY, USA).

### 2.5 Role of the funding organization

Two members of the Dutch PD Patient Association made a contribution to the design of the study. The funding bodies and Dezzel Media B.V. had no role in the collection, analysis, and interpretation of data, writing the manuscript, or the decision to submit the manuscript for publication.

## 3 Results

### 3.1 Participants

We enrolled 140 PD patients between September 15th 2017 and May 23rd 2019 with two-year follow-up assessments until August 9th 2021 (see Supplementary Figure S1). Four participants were wrongfully enrolled and therefore excluded from the analyses. The T3 (n = 109) and T4 (n = 103) data showed a subtle attrition bias as the follow-up subsamples consisted of marginally younger and cognitively better performing participants (see Table 1 and Supplementary Table S2).

**Table 1:** Sample description of the intention-to-treat population.

	Baseline – T0		1y follow-up – T3			2y follow-up – T4		
	Active control (n = 68)	Cognitive training (n = 68)	Active control (n = 53)	Cognitive training (n = 56)	p-value	Active control (n = 51)	Cognitive training (n = 52)	p-value
Sex (N Female (%))	21 (31%)	33 (49%)	18 (34%)	27 (48%)	0.173	17 (33%)	26 (50%)	0.111
Age (years)	62.9 (7.0)	62.9 (8.1)	63.7 (6.6)	63.8 (8.3)	0.805	65.1 (6.4)	64.4 (8.2)	0.786
Education (years)	16.7 (4.4)	15.5 (3.3)	16.9 (4.4)	15.6 (3.5)	0.110	16.6 (4.5)	15.8 (3.5)	0.274
Education classification (median [range])^a^	6 [3-7]	6 [3-7]	6 [4–7]	6 [3-7]	0.850	6 [3-7]	6 [3-7]	0.948
Disease duration (years, median [range])	5 [1–26]	5 [0–22]	6 [2–27]	6 [1–23]	0.900	6 [3–28]	7 [2–24]	0.487
UPDRS-III	21.0 (9.5)	20.2 (8.3)	19.3 (8.9)	19.0 (10.6)	0.938	23.3 (12.3)	21.4 (12.1)	0.401
Hoehn & Yahr stage (N (%))					0.429			0.751
1	5 (7.4%)	4 (5.9%)	5 (9.4%)	5 (8.9%)	–	3 (5.9%)	5 (9.6%)	–
1.5	2 (2.9%)	7 (10.3%)	5 (9.4%)	2 (3.6%)	–	2 (3.9%)	2 (3.8%)	–
2	34 (50.0%)	28 (41.2%)	26 (49.1%)	27 (48.2%)	–	24 (47.1%)	28 (53.8%)	–
2.5	18 (26.5%)	18 (26.5%)	8 (15.1%)	15 (26.8%)	–	9 (17.6%)	8 (15.4%)	–
3	9 (13.2%)	11 (16.2%)	9 (17.0%)	6 (10.7%)	–	10 (19.6%)	5 (9.6%)	–
4	0 (0.0%)	0 (0.0%)	0 (0.0%)	0 (0.0%)	–	3 (5.9%)	2 (3.8%)	–
5	0 (0.0%)	0 (0.0%)	0 (0.0%)	1 (1.8%)	–	0 (0.0%)	0 (0.0%)	–
Medication use								
LEDD (median [range])	650 [0–2100]	737 [0–1665]	758 [0–2221]	800 [80–2983]	0.627	814 [0–2219]	772 [0–2900]	0.843
Antidepressants (N (%))	7 (10.3%)	9 (13.2%)	3 (5.7%)	5 (9.1%)	0.717	4 (8.0%)	5 (9.6%)	1.000
Cholinesterase inhibitors (N (%))	2 (2.9%)	1 (1.5%)	2 (3.8%)	2 (3.8%)	1.000	0 (0%)	4 (7.7%)	0.118
Antipsychotics (N (%))	0 (0%)	2 (2.9%)	0 (0%)	1 (1.8%)	1.000	1 (2.0%)	0 (0%)	0.490
Benzodiazepine agonists (N (%))	12 (17.7%)	7 (10.3%)	4 (7.5%)	1 (1.8%)		4 (7.8%)	2 (3.8%)	
MoCA	25.9 (2.3)	26.3 (2.0)	26.0 (2.5)	26.7 (2.5)	*	25.5 (2.6)	26.0 (2.7)	*
Cognitive status (N (%))					0.960			0.581
Normal cognition	13 (19.1%)	15 (22.1%)	16 (30.2%)	19 (33.9%)	–	12 (23.5%)	17 (32.7%)	–
Single-domain MCI	7 (10.3%)	9 (13.2%)	4 (7.5%)	3 (5.4%)	–	3 (5.9%)	5 (9.6%)	–
Multi-domain MCI	35 (51.5%)	34 (50.0%)	25 (47.2%)	25 (44.6%)	–	27 (52.9%)	24 (46.2%)	–
PD dementia	13 (19.1%)	10 (14.7%)	8 (15.1%)	9 (16.1%)	–	9 (17.6%)	6 (11.5%)	–
BDI	7.87 (4.1)	8.21 (4.0)	10.3 (6.5)	9.0 (4.4)	*	11.4 (5.8)	9.0 (4.7)	*
QUIP-RS	19.2 (12.7)	15.8 (12.8)	21.3 (13.3)	18.4 (12.4)	*	22.6 (13.3)	18.3 (12.9)	*
PAS	10.5 (6.8)	10.3 (6.6)	12.5 (8.5)	10.8 (6.0)	*	12.8 (7.9)	11.4 (7.0)	*
AS	13.4 (4.5)	13.2 (4.5)	14.6 (5.9)	14.2 (5.2)	*	15.6 (6.1)	13.9 (4.9)	*
PD-CFRS (median [range])	9.0 [3.3–22]	7.0 [3.3–19]	7.0 [0–23]	7.3 [0–17.5]	*	7.6 [0–22]	7.1 [0–20.4]	*
Compliance (%, median [range])	100 [25–100]	100 [39–100]	–	–	–	–	–	–
T0-to-FU interval (days)	–	–	441 (20)	439 (19)	0.533	819 (49)	816 (43)	0.679
CRIq (n = 113) - Total	–	–	–	–	–	127.1 (19.8)	126.9 (15.6)	0.944
Education	–	–	–	–	–	128.3 (22.8)	126.3 (14.8)	0.578
Work	–	–	–	–	–	114.3 (19.3)	114.3 (16.1)	0.977
Leisure	–	–	–	–	–	118.9 (22.1)	120.3 (20.3)	0.729

Data are mean (SD) unless otherwise specified. ^a^According to Verhage education classification.^29^ *Secondary outcome variables were analyzed using linear mixed-models and described elsewhere in the manuscript. *Abbreviations*: AS = Apathy Scale; BDI = Beck Depression Inventory; PAS = Parkinson Anxiety Scale; PD-CFRS = Parkinson’s Disease – Cognitive Functional Rating Scale; LEDD = Levodopa equivalent daily dosage; MCI = mild cognitive impairment; MoCA = Montreal Cognitive Assessment; QUIP-RS = Questionnaire for Impulsive-Compulsive Disorders in Parkinson's Disease – Rating Scale; UPDRS = Unified Parkinson’s Disease Rating Scale.

### 3.2 Neuropsychological test outcomes

**Fig. 1:**
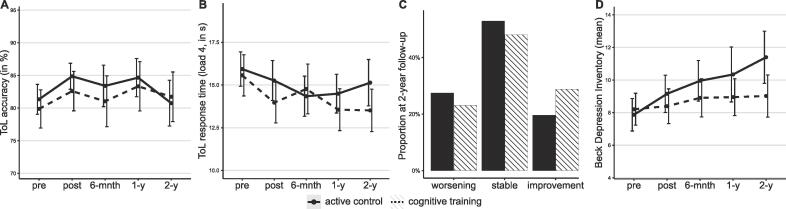
Development of cognitive function (**A**, **B**, **C**) and depressive symptoms (**D**) from baseline to follow-up. **A** and **B** show the performance on the Tower of London (ToL) accuracy and response time. In **C**, worsening represents participants that converted to a worse cognitive status from baseline to 2-year follow-up while improvement represents the opposite. Error bars represent 95% CI.

Statistics of all mixed-model analyses are presented in Supplementary Table S3. No group differences were found on the accuracy of the Tower of London task across task-loads at one- and two-year follow-up, corrected for baseline performance and demographic covariates: T3 – B[SE]: -0.053 [0.115], 95% CI: -0.279 to 0.173, p = .643; T4 – B[SE]: 0.039 [0.116], 95% CI: -0.189 to 0.267, p = .736 (see Fig. 1A). On the Tower of London response time also no group differences were present at T3 and T4: T3 – B[SE]: -0.089 [0.095], 95% CI: -0.277 to 0.100, p = .354, T4 – B[SE]: -0.097 [0.096], 95% CI: -0.286 to 0.093, p = .315. Notably, there were no group differences at either follow-up time-point on difficulty load 4 of the Tower of London, that showed group differences at the post-intervention assessment (T1) [5] (see Fig. 1B). None of the other individual task load accuracies or response times differed between groups at each of the follow-up time points and there were no differences in subjective cognitive complaints.

Regarding the other neuropsychological tests, no group differences were present at one- and two-year follow-up (Supplementary Table S4). At T3, the active control group showed marginally better performance on the Boston Naming Test (B[SE] -0.94, [0.36], p = .01); an effect that disappeared at T4 and is presumably related to a ceiling effect on this task. Conversely, the cognitive training group showed better Stroop Color Word Test interference performance at T4 (B[SE]: −12.4 [4.77], p = .009), which seemed partly driven by one extreme score in the active control group (estimates after exclusion: B[SE]: −7.7 [4.3], p = .072). These isolated group differences did not survive correction for multiple comparisons.

Post-hoc analysis of solely the PD-MCI subgroup (active control: n = 43 vs. cognitive training: n = 42) did not show effects specific for this subgroup as reported in Supplementary Table S5.

### 3.3 Classification of cognitive status

There was no association between condition and conversion of cognitive status at T3, T4 and between T3 and T4 (T4: see Fig. 1C). Participants in the active control condition were equally likely to show conversion to a worse cognitive status as those in the cognitive training condition: T3 relative to T0 – OR (95% CI): 0.96 (0.38 to 2.41); T4 relative to T0 – OR (95% CI): 1.26 (0.52 to 3.08); T4 relative to T3 – OR (95% CI): 1.23 (0.46 to 3.25).

### 3.4 Exploratory psychiatric outcomes

At T4, the cognitive training group had lower mean BDI scores compared with the active control condition, after correcting for baseline values: B[SE]: −2.50 [0.86], 95% CI: −4.19 to -0.81, p = .004 (Fig. 1D). Parkinson Anxiety Scale and Apathy Scale scores were also lower in the cognitive training group at T4, although not statistically significant. At T3, group differences concerning the BDI and Parkinson Anxiety Scale were comparable to T4 but not statistically significant (see Supplementary Table S3).

## 4 Discussion

This study did not replicate previously reported long-term positive effects of cognitive training up to eighteen months. Although we previously showed positive effects of our cognitive training intervention on processing speed directly after training [5], no effects were present at follow-up relative to an active control condition. Rate of conversion to PD-MCI and PD-D was similar in both study groups, suggesting no measurable preventive effect of our cognitive training on cognitive decline.

One earlier study on the long-term effects of cognitive training in PD lacked comparison with a control condition limiting the differentiation between cognitive training effects and test–retest effects [2], while the other study used a supervised, group-based training condition [3]. The results of current trial [5] – in line with a recent *meta*-analysis [1] – show evidence for the direct but short lasting effects of cognitive training in improving performance on cognitive tasks. For the clinical applicability of cognitive training, however, intervention modifications are required to improve far transfer effects to subjective experience and prolong effects to the long term, and there is a need for knowledge on the optimal length and frequency of cognitive training in PD. Booster sessions are a viable option to increase the efficacy of cognitive training and its long-term effects [12], [13]. Our results additionally suggest that the *BrainGymmer* cognitive training may have a beneficial effect on mood. Given the conflicting data on the effects of cognitive training on mood in the present and previous studies [3], [14], this issue warrants further study.

A limitation of current study was the heterogeneous study population – with a subset of participants showing cognitive deficits suggestive of PD-D despite exclusion criteria – possibly diluting the intervention effects. Secondly, our study lacked a passive control condition. Consequently, we were not able to differentiate between positive effects in both intervention groups versus no long-term effects of cognitive training. Although it is generally advised to use active comparators [15], an additional wait-list control group could have informed on this differentiation. Additionally, there was an attrition bias, most notably at T4. The attrition bias and test–retest effects may have attenuated measurable cognitive decline and at T4 about 25% of assessments were missed – in part due to governmental COVID-19 regulations. However, the study sample seems (on average) to have been relatively stable over the two-year period concerning other parameters of PD progression, which is not uncommon in naturalistic follow-up studies [16], [17]. Strengths of this study include the relatively large sample size, excellent intervention compliance and extensive neuropsychological assessment enabling us to use level II criteria for PD-MCI and probable PD-D.

In conclusion, this study found no evidence for long-term improvements of cognitive function or a delay of cognitive decline following an eight-week computerized cognitive training with *BrainGymmer*. Future studies are necessary to work towards a best practice for cognitive training methods, including definition of an optimal training length and dosage, and assessment of the potential of additional booster sessions to retain positive effects.

## Declaration of Competing Interest

The authors declare that they have no known competing financial interests or personal relationships that could have appeared to influence the work reported in this paper.
