# Supplementary material for: Long-term effects of cognitive training in Parkinson’s disease: A randomized, controlled trial
Source: Clin Park Relat Disord. 2023 Jun 7;9:100204. doi: 10.1016/j.prdoa.2023.100204 (PMC10724826; doi:10.1016/j.prdoa.2023.100204)
Supplement: Supplementary data 1 [file mmc1.pdf]

Supplementary material

Long-term effects of eight-week computerized cognitive training in Parkinson's disease: a randomized, controlled trial

Authors

Tim D. van Balkom PhD, Odile A. van den Heuvel MD PhD, Henk W. Berendse MD PhD, Ysbrand D. van der Werf PhD, Rob H. Hagen MSc, Tanja Berk MSc, and Chris Vriend PhD

Supplementary Table S1 Overview of all study assessments at all time-points.

**Time-point**	**T-2**	**T-1**	**T0**	**T1**	**T2**	**T3**	**T4**
**Pre-screening**							
Informed consent for pre-screening	X						
SAGE[1]	X						
PD-CFRS[2]	X			X	X	X	X
MRI safety screening	X						
Alcohol abuse screening (CAGE-AID) [3, 4]	X						
**Eligibility screening**							
MoCA[5]		X		X	X	X	X
ICD diagnostic criteria		X		X			
SAPS-PD[†,6]		X					
BDI[7]		X		X	X	X	X
Hoehn & Yahr stage[8]		X				X	X
**Enrolment and allocation**			X				
**Intervention**							
Cognitive training				←→			
Active control condition				←→			
**Assessments**							
Neuropsychological assessment							
A Tower of London[9]			X	X	X	X	X
Pentagon copy[10, 11]			X	X	X	X	X
A/B Stroop Color Word Test[12]			X	X	X	X	X
A COWAT ('letter fluency') [†,13]			X	X	X	X	X
B WAIS-III digit span[14]			X	X	X	X	X
C Rey Auditory Verbal Learning Test[‡,15]			X	X	X	X	X
C Location Learning Test[#,16]			X	X	X	X	X
D Boston naming test[17]			X	X	X	X	X
D Category fluency[18]			X	X	X	X	X
E Rey Complex Figure Test[19]			X	X	X	X	X
E Visual Form Discrimination Test[20]			X	X	X	X	X
Questionnaires and interviews							
CFQ[21]			X	X	X	X	X
Apathy scale[22]			X	X	X	X	X
Parkinson anxiety scale[23]			X	X	X	X	X
QUIP-RS[24]			X	X	X	X	X
NZPAQ-SF[25]			X	X	X	X	X
Credibility/expectancy questionnaire[26]			X				
Cognitive Reserve Index questionnaire[27]							X
Motor symptom assessments							
UPDRS-III - motor score[28]			X			X	X
Medication use							
Levodopa equivalent daily dosage[29]			X	X	X	X	X
*Neuroimaging**							
MP-RAGE			X	X			
3D PSIR			X	X			
fMRI - resting state			X	X			
DTI			X	X			

*in a subsample of N = 80.
Parallel forms of the same test are used at consecutive visits if available: [†]Three parallel forms; [‡]Two parallel forms; [#]One parallel form.
Cognitive domains: [A]Executive function, [B]Attention and working memory, [C]Memory, [D]Language, [E]Visuospatial.
Abbreviations: BDI = Beck depression inventory; CFQ = Cognitive Failures Questionnaire; COWAT = Controlled Oral Word Association Test; DTI = diffusion tensor imaging; MP RAGE= magnetization-prepared 180 degrees radio-frequency pulses and rapid gradient-echo; MoCA = Montreal Cognitive Assessment; (f)MRI = (functional) magnetic resonance imaging; NZPAQ-SF = New Zealand Physical Activity Questionnaire – Short Form; PD-CFRS = Parkinson's Disease – Cognitive Functional Rating Scale; PSIR = phase-sensitive inversion recovery; QPE = Questionnaire for Psychotic Experiences; QUIP-RS = Questionnaire for Impulsive-Compulsive Disorders in Parkinson's Disease – Rating Scale; SAPS-PD: Scale for Assessment of Positive Symptoms for Parkinson's disease; UPDRS = Unified Parkinson's Disease Rating Scale; WAIS = Wechsler Adult Intelligence Scale.

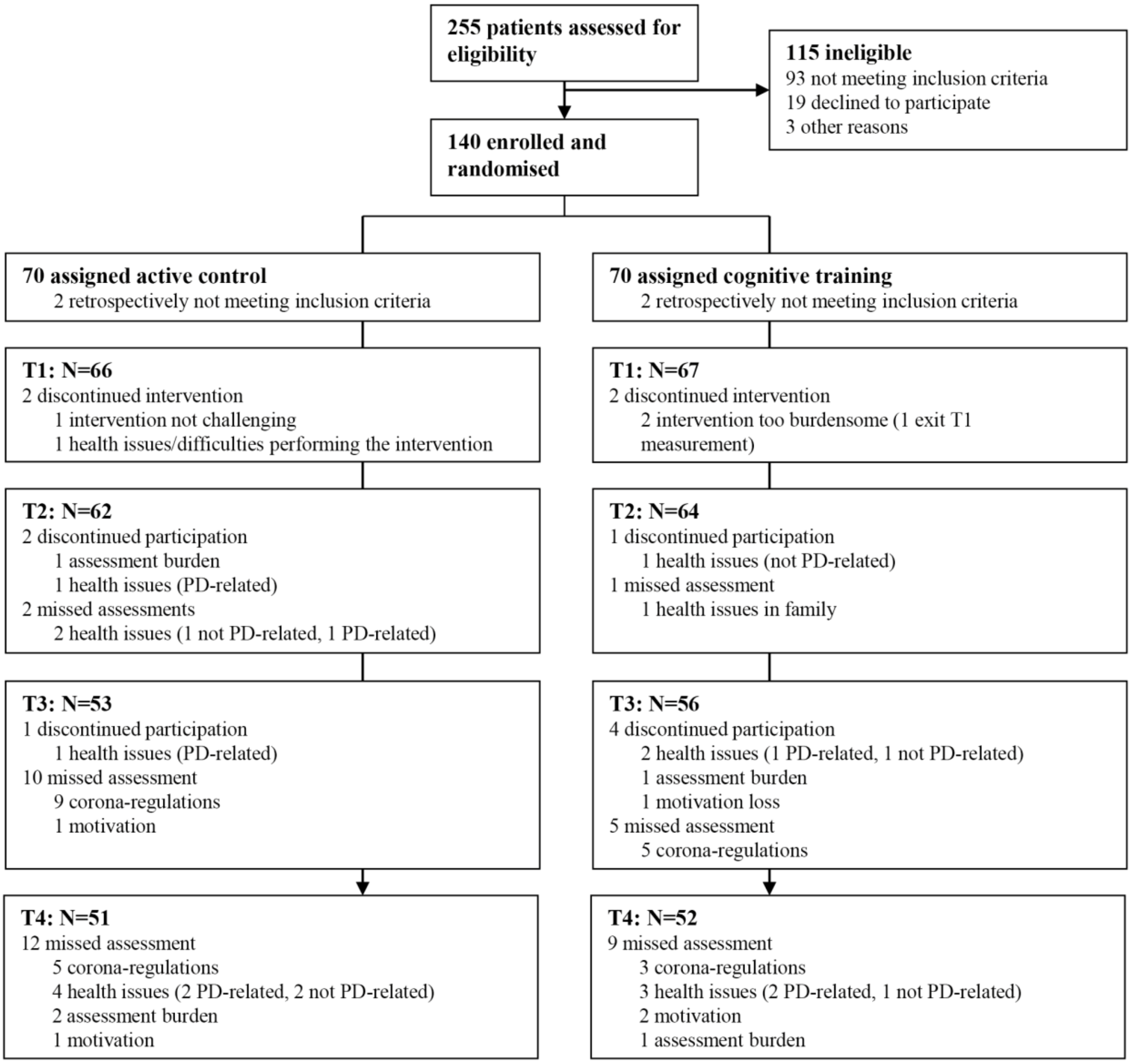

Supplementary Figure S1 CONSORT flowchart.

Supplementary Table S2 Comparison between samples that did and did not undergo the one-year (T3) or two-year (T4) follow-up assessment.

	T3 assessment taken			T4 assessment taken		
	**No (n=27)**	**Yes (n=109)**	**p**	**No (n=33)**	**Yes (n=103)**	**p**
**Age**	64.3 (8.0)	62.5 (7.4)	0.269	64.0 (8.1)	62.5 (7.4)	0.331
**Sex (N (%) Female)**	9 (33.3%)	45 (41.3%)	0.515	11 (33.3%)	43 (41.7%)	0.421
**Education classification**	6 [3-7]	6 [3-7]	0.550	6 [5-7]	6 [3-7]	0.811
**Years of education**	15.6 (3.7)	16.2 (4.0)	0.461	15.8 (3.5)	16.2 (4.1)	0.624
**Disease duration**	6.6 (3.9)	6.5 (5.3)	0.929	7.9 (4.4)	6.1 (5.1)	*0.066*
**UPDRS-III motor score**	20.0 (9.9)	20.7 (8.7)	0.735	20.7 (10.9)	20.5 (8.2)	0.937
**Hoehn & Yahr stage**			0.931			0.556
1	3 (11.1%)	6 (5.5%)		4 (12.1%)	5 (4.9%)	
1.5	1 (3.7%)	8 (7.3%)		1 (3%)	8 (7.8%)	
2	11 (40.7%)	51 (46.8%)		12 (36.4%)	50 (48.5%)	
2.5	8 (29.6%)	28 (25.7%)		10 (30.3%)	26 (25.2%)	
3	4 (14.8%)	16 (14.7%)		6 (18.2%)	14 (13.6%)	
**LEDD**	600 [210-2100]	724 [0-2081]	0.480	995 [300-2100]	620 [0-2081]	**0.016**
**MoCA**	25.4 (2.1)	26.3 (2.1)	*0.068*	25.6 (2.3)	26.3 (2.1)	0.115
**Average normalized cognitive function**	44.6 (3.5)	46.5 (5.1)	*0.065*	44.6 (5.2)	46.6 (4.7)	**0.048**
**Cognitive classification**			0.178			**0.022**
normal cognition	3 (11.1%)	25 (22.9%)		4 (12.1%)	24 (23.3%)	
single-domain MCI	0 (0%)	16 (14.7%)		2 (6.1%)	14 (13.6%)	
multi-domain MCI	21 (77.8%)	48 (44%)		18 (54.5%)	51 (49.5%)	
dementia	3 (11.1%)	20 (18.3%)		9 (27.3%)	14 (13.6%)	
**PD-CFRS**	9.7 (4.3)	8.8 (4.3)	0.284	9.2 (4.0)	8.9 (4.4)	0.688
**CFQ**	37.8 (9.5)	38.8 (11.3)	0.661	38.2 (11.0)	38.8 (11.0)	0.784
**BDI**	6.6 (2.6)	8.4 (4.3)	**0.034**	7.4 (4.2)	8.2 (4.0)	0.322
**PAS**	11.1 (6.2)	10.2 (6.8)	0.531	10.5 (6.1)	10.4 (6.9)	0.946
**AS**	12.4 (4.4)	13.5 (4.5)	0.254	13.3 (4.4)	13.3 (4.5)	0.981
**QUIP-RS**	19.1 (12.2)	17.0 (13.0)	0.452	18.5 (13.2)	17.0 (12.7)	0.581

Abbreviations: AES = Apathy Scale; BDI = Beck Depression Inventory; CFQ = Cognitive Failures Questionnaire; LEDD = Levodopa equivalent daily dosage; MoCA = Montreal Cognitive Assessment; PAS = Parkinson Anxiety Scale; PD-CFRS = Parkinson's Disease – Cognitive Functional Rating Scale; QUIP-RS = Questionnaire for Impulsive-Compulsive Disorders in Parkinson's Disease – Rating Scale; UPDRS = Unified Parkinson's Disease Rating Scale.

Supplementary Table S3 Group differences from the multivariate and univariate linear mixed-model analyses on the Tower of London (primary study outcome) and subjective cognitive complaints and psychiatric symptom questionnaires.

	Baseline		One-year follow-up		Group difference[a]			Two-year follow-up		Group difference[a]		
	Active control M (SD)	Cognitive training M (SD)	Active control M (SD)	Cognitive training M (SD)	B [SE]	95% CI	*P* value	Active control M (SD)	Cognitive training M (SD)	B [SE]	95% CI	*P* value
**Neuropsychological test outcomes**												
Overall ToL accuracy (%)	82.5 (7.9)	81.0 (9.1)	84.7 (10.5)	83.3 (14.0)	-0.053 [0.115]	-0.279 to 0.173	0.643	82.1 (10.6)	82.7 (11.7)	0.039 [0.116]	-0.189 to 0.267	0.736
S1	96.3 (5.2)	96.1 (5.9)	97.4 (5.2)	97.1 (5.2)	-0.146 [0.173]	-0.486 to 0.194	0.399	95.8 (6.1)	96.5 (5.6)	0.038 [0.176]	-0.307 to 0.382	0.829
S2	91.9 (8.8)	90.8 (9.0)	91.9 (10.9)	90.7 (9.5)	-0.120 [0.173]	-0.460 to 0.219	0.487	90.3 (10.6)	90.4 (10.8)	0.025 [0.176]	-0.319 to 0.370	0.885
S3	88.0 (11.1)	86.5 (12.1)	88.0 (13.0)	89.1 (11.2)	0.027 [0.173]	-0.313 to 0.367	0.876	85.7 (11.8)	86.1 (13.4)	0.036 [0.176]	-0.309 to 0.380	0.839
S4	76.2 (13.6)	74.9 (15.3)	78.2 (16.1)	80.4 (18.4)	0.065 [0.173]	-0.275 to 0.404	0.708	75.7 (15.1)	77.8 (18.0)	0.113 [0.176]	-0.232 to 0.457	0.521
S5	60.2 (20.4)	56.8 (19.7)	67.8 (20.9)	66.6 (23.3)	-0.093 [0.173]	-0.433 to 0.247	0.591	63.1 (25.4)	62.7 (21.9)	-0.018 [0.176]	-0.363 to 0.326	0.917
Overall ToL reaction time (s)[b,c]	12.6 (2.8)	12.5 (3.2)	11.8 (2.8)	11.2 (3.2)	-0.089 [0.095]	-0.277 to 0.100	0.354	12.0 (3.2)	11.0 (3.2)	-0.097 [0.096]	-0.286 to 0.093	0.315
S1	6.3 (1.9)	5.9 (1.7)	6.0 (2.1)	5.4 (2.0)	-0.072 [0.132]	-0.330 to 0.187	0.587	6.1 (2.2)	5.6 (2.6)	-0.056 [0.133]	-0.318 to 0.205	0.672
S2	8.3 (2.6)	8.2 (2.9)	7.7 (2.5)	7.1 (3.1)	-0.122 [0.132]	-0.380 to 0.137	0.355	7.9 (3.0)	7.4 (3.4)	-0.086 [0.133]	-0.348 to 0.176	0.519
S3	10.9 (2.7)	11.1 (3.5)	10.6 (3.1)	9.5 (3.2)	-0.169 [0.132]	-0.427 to 0.090	0.200	11.0 (3.9)	9.8 (4.1)	-0.232 [0.133]	-0.494 to 0.029	0.082
S4	15.6 (3.8)	15.6 (4.8)	14.5 (4.1)	13.5 (4.5)	-0.132 [0.132]	-0.390 to 0.127	0.317	14.8 (4.5)	13.6 (4.4)	-0.151 [0.133]	-0.413 to 0.111	0.258
S5	22.0 (4.9)	21.6 (4.8)	20.3 (4.9)	19.8 (4.8)	0.053 [0.132]	-0.205 to 0.312	0.687	20.0 (5.1)	19.2 (4.8)	0.045 [0.133]	-0.217 to 0.307	0.735
**Subjective cognitive complaints**												
PD-CFRS[b]	9.7 (4.6)	8.2 (3.9)	7.9 (4.8)	7.5 (4.8)	0.29 [0.77]	-1.22 to 1.79	0.708	9.0 (5.2)	7.7 (4.6)	-0.39 [0.82]	-2.00 to 1.21	0.630
CFQ[b]	38.6 (11.4)	38.6 (10.5)	39.1 (12.7)	37.2 (11.4)	-0.84 [1.63]	-4.04 to 2.36	0.606	39.2 (12.2)	40.2 (12.4)	2.04 [1.72]	-1.34 to 5.43	0.236
**Psychiatric symptom questionnaires**												
BDI[b]	7.9 (4.1)	8.2 (4.0)	10.3 (6.5)	9.0 (4.4)	-1.65 [0.81]	-3.25 to -0.06	0.042	11.4 (5.8)	9.0 (4.7)	-2.50 [0.86]	-4.19 to - 0.81	0.004
PAS[b]	10.5 (6.8)	10.3 (6.6)	12.5 (8.5)	10.8 (6.0)	-1.75 [1.03]	-3.76 to 0.27	0.089	12.8 (7.9)	11.4 (7.0)	-1.87 [1.09]	-4.02 to 0.27	0.087
AS[b]	13.4 (4.5)	13.2 (4.5)	14.6 (5.9)	14.2 (5.2)	-0.37 [0.68]	-1.70 to 0.96	0.581	15.6 (6.1)	13.9 (4.9)	-1.32 [0.72]	-2.73 to 0.10	0.067
QUIP-RS[b]	19.2 (12.7)	15.8 (12.8)	21.3 (13.3)	18.4 (12.4)	1.08 [1.93]	-2.70 to 4.87	0.574	22.6 (13.3)	18.3 (12.9)	-1.52 [2.04]	-5.53 to 2.48	0.455

[a]Corrected for baseline score and age, sex and education in years; [b]Lower is better; negative estimates indicate effects in favor of CT; [c]Reaction time of correct responses. Abbreviations: AS = Apathy Scale; BDI = Beck Depression Inventory; CFQ = Cognitive Failures Questionnaire; PAS = Parkinson Anxiety Scale; PD-CFRS = Parkinson's disease – Cognitive Functional Rating Scale; QUIP-RS = Questionnaire for Impulsive-Compulsive Disorders in Parkinson's Disease – Rating Scale; ToL = Tower of London.

Supplementary Table S4 Group differences from the univariate linear mixed-model analyses on the neuropsychological assessment at one-year (T3) and two-year (T4) follow-up.

	Baseline		One-year follow-up		Group difference[a]			Two-year follow-up		Group difference[a]		
	Active control M (SD)	Cognitive training M (SD)	Active control M (SD)	Cognitive training M (SD)	B [SE]	95% CI	*P* value	Active control M (SD)	Cognitive training M (SD)	B [SE]	95% CI	*P* value
**MoCA**	25.9 (2.3)	26.3 (2.0)	26.0 (2.5)	26.7 (2.5)	0.45 [0.39]	-0.32 to 1.21	0.252	25.5 (2.6)	26.0 (2.7)	0.25 [0.40]	-0.54 to 1.04	0.538
**SCWT card I (s)[b]**	56.0 (14.9)	53.9 (10.7)	51.4 (12.2)	49.7 (10.0)	0.74 [1.60]	-2.39 to 3.88	0.642	55.4 (18.9)	50.7 (10.7)	-1.32 [1.65]	-4.57 to 1.92	0.422
**SCWT card II (s)[b]**	68.0 (13.6)	68.0 (17.9)	65.8 (16.6)	64.0 (17.1)	-1.77 [1.83]	-5.37 to 1.84	0.335	69.6 (18.7)	64.7 (17.1)	-2.26 [1.89]	-5.98 to 1.46	0.233
**SCWT card III (s)[b]**	107.1 (33.3)	114.8 (49.0)	103.3 (30.5)	99.7 (31.7)	-5.12 [4.64]	-14.23 to 3.99	0.270	111.8 (54.0)	101.3 (36.2)	-12.43 [4.77]	-21.80 to -3.06	0.009
**COWAT letter fluency**	38.9 (11.9)	37.8 (10.6)	43.6 (11.7)	41.3 (11.3)	-1.58 [1.43]	-4.39 to 1.24	0.272	43.8 (13.2)	45.0 (12.3)	1.46 [1.47]	-1.43 to 4.36	0.321
**Category fluency**	23.6 (5.3)	22.1 (5.1)	23.0 (6.5)	22.3 (5.8)	0.30 [0.84]	-1.35 to 1.95	0.722	20.4 (7.1)	21.1 (5.3)	1.15 [0.87]	-0.56 to 2.85	0.187
**RCFT**	30.1 (3.6)	31.0 (3.6)	30.3 (4.6)	30.6 (4.8)	-0.08 [0.60]	-1.26 to 1.09	0.889	30.5 (4.6)	31.2 (4.9)	-0.21 [0.61]	-1.42 to 1.00	0.729
**RAVLT direct recall**	41.4 (11.7)	41.1 (10.8)	48.8 (10.8)	49.3 (10.4)	1.04 [1.43]	-1.76 to 3.85	0.465	42.3 (12.2)	43.1 (12.9)	0.76 [1.46]	-2.11 to 3.63	0.603
**RAVLT delayed recall**	8.3 (3.5)	8.2 (3.5)	10.2 (2.9)	10.5 (3.6)	0.23 [0.46]	-0.68 to 1.14	0.616	7.9 (3.3)	8.7 (3.7)	0.40 [0.47]	-0.53 to 1.34	0.394
**RAVLT recognition**	28.3 (2.2)	28.4 (1.9)	28.7 (1.8)	28.8 (2.1)	0.12 [0.34]	-0.56 to 0.79	0.730	28.3 (1.9)	28.2 (2.7)	-0.11 [0.35]	-0.80 to 0.58	0.752
**Digit span forward score**	9.5 (1.8)	9.4 (1.6)	9.8 (2.0)	9.7 (1.7)	-0.01 [0.27]	-0.55 to 0.52	0.958	9.6 (1.9)	9.8 (1.8)	0.20 [0.28]	-0.35 to 0.75	0.483
**Digit span backward score**	7.3 (1.6)	7.0 (1.6)	7.4 (1.8)	7.2 (1.5)	0.00 [0.27]	-0.53 to 0.52	0.986	7.2 (1.8)	7.0 (1.2)	0.02 [0.27]	-0.52 to 0.56	0.945
**LLT direct recall[b]**	21.0 (16.0)	21.5 (21.8)	20.9 (18.7)	19.0 (15.8)	-2.86 [2.90]	-8.56 to 2.84	0.325	24.7 (24.3)	19.0 (17.5)	-5.08 [3.00]	-10.97 to 0.82	0.091
**LLT delayed recall[b]**	1.8 (3.0)	1.8 (4.7)	1.6 (4.0)	1.8 (3.1)	0.15 [0.51]	-0.85 to 1.15	0.765	1.9 (3.2)	1.7 (3.4)	-0.07 [0.52]	-1.10 to 0.96	0.894
**BNT**	55.4 (3.0)	55.4 (3.4)	57.4 (2.1)	56.4 (3.5)	-0.94 [0.36]	-1.66 to -0.23	0.010	56.7 (3.0)	56.7 (3.0)	-0.06 [0.37]	-0.79 to 0.68	0.878
**BVFDT**	29.8 (2.5)	30.1 (1.8)	29.9 (2.6)	30.1 (2.2)	0.10 [0.40]	-0.69 to 0.89	0.806	30.2 (2.0)	30.6 (1.8)	0.20 [0.41]	-0.61 to 1.01	0.625

[a]Corrected for baseline score and age, sex and education in years; [b]Lower is better - negative estimates indicate effects in favor of CT.

Abbreviations: BNT = Boston Naming Test, BVFDT = Benton Visual Form Discrimination Test, COWAT = Controlled Oral Word Association Test, LLT = Location Learning Test, RAVLT = Rey Auditory Verbal Learning Test, RCFT = Rey Complex Figure Test, SCWT = Stroop Color Word Test.

Supplementary Table S5 Group differences <u>in the PD-MCI subgroup</u> (n=85) from the univariate linear mixed-model analyses on the neuropsychological assessment at one-year (T3) and two-year (T4) follow-up.

	**Baseline**		**T3: One-year follow-up**		**Group difference[a]**			**T4: Two-year follow-up**		**Group difference[a]**		
	Active control M (SD)	Cognitive training M (SD)	Active control M (SD)	Cognitive training M (SD)	B [SE]	95% CI	*P* value	Active control M (SD)	Cognitive training M (SD)	B [SE]	95% CI	*P* value
**MoCA**	25.8 (2.2)	26.4 (1.7)	26.1 (2.3)	26.7 (2.1)	0.58 [0.51]	-0.42 to 1.57	0.257	25.4 (2.4)	25.9 (2.6)	0.43 [0.50]	-0.55 to 1.41	0.388
**SCWT card I (s)[b]**	55.4 (12.2)	54.4 (8.0)	50.3 (9.4)	50.6 (8.3)	1.48 [2.03]	-2.51 to 5.47	0.466	53.1 (11.5)	53.2 (10.2)	1.06 [2.01]	-2.89 to 5.01	0.598
**SCWT card II (s)[b]**	68.2 (11.0)	69.3 (12.2)	63.4 (9.2)	66.3 (14.3)	1.95 [2.08]	-2.14 to 6.04	0.348	67.7 (11.8)	68.8 (17.9)	0.35 [2.06]	-3.71 to 4.41	0.865
**SCWT card III (s)[b]**	106.3 (27.8)	119.9 (52.1)	99.6 (25.0)	107.1 (32.0)	2.18 [5.47]	-8.58 to 12.94	0.690	103.8 (37.2)	111.8 (39.1)	1.44 [5.44]	-9.26 to 12.14	0.792
**COWAT letter fluency**	38.1 (10.0)	38.1 (8.2)	40.7 (8.8)	41.5 (10.6)	0.68 [1.79]	-2.85 to 4.21	0.704	41.9 (11.0)	43.4 (11.6)	1.82 [1.78]	-1.68 to 5.32	0.307
**Category fluency**	23.2 (5.1)	21.5 (5.3)	23.0 (5.2)	21.7 (5.0)	0.40 [1.10]	-1.75 to 2.56	0.714	20.0 (6.9)	19.7 (5.5)	0.98 [1.09]	-1.16 to 3.12	0.367
**RCFT**	30.4 (3.0)	31.2 (2.9)	30.9 (3.4)	31.5 (3.8)	0.63 [0.74]	-0.81 to 2.08	0.389	30.6 (3.4)	31.5 (4.5)	0.60 [0.73]	-0.83 to 2.03	0.408
**RAVLT direct recall**	40.8 (11.3)	39.5 (9.7)	48.5 (9.8)	48.1 (8.6)	2.02 [1.82]	-1.57 to 5.60	0.269	40.7 (11.5)	41.6 (13.0)	2.50 [1.77]	-0.99 to 6.00	0.159
**RAVLT delayed recall**	8.0 (3.3)	7.6 (3.0)	9.9 (2.9)	10.4 (3.1)	1.06 [0.58]	-0.07 to 2.20	0.066	7.5 (3.0)	8.3 (3.3)	1.15 [0.57]	0.04 to 2.27	0.043
**RAVLT recognition**	28.0 (2.5)	28.3 (2.1)	28.5 (2.0)	28.5 (2.6)	0.38 [0.46]	-0.53 to 1.29	0.415	28.2 (1.8)	27.8 (2.9)	-0.12 [0.46]	-1.02 to 0.78	0.794
**Digit span forward score**	9.3 (1.4)	9.4 (1.4)	9.7 (1.9)	9.7 (1.7)	-0.03 [0.34]	-0.71 to 0.65	0.938	9.4 (1.7)	9.6 (1.6)	0.15 [0.34]	-0.53 to 0.82	0.671
**Digit span backward score**	7.1 (1.5)	7.0 (1.5)	7.2 (1.8)	7.0 (1.3)	0.07 [0.33]	-0.59 to 0.73	0.828	7.3 (1.6)	7.2 (1.2)	0.11 [0.33]	-0.54 to 0.76	0.738
**LLT direct recall[b]**	23.6 (17.7)	23.5 (22.5)	22.5 (19.9)	20.1 (15.5)	-6.96 [3.89]	-14.62 to 0.69	0.074	23.9 (24.6)	21.9 (18.8)	-5.91 [3.85]	-13.48 to 1.67	0.126
**LLT delayed recall[b]**	2.3 (3.6)	2.1 (5.1)	1.8 (4.1)	1.9 (3.0)	-0.63 [0.69]	-1.99 to 0.73	0.363	1.8 (3.0)	2.0 (3.8)	-0.40 [0.68]	-1.74 to 0.94	0.558
**BNT**	55.2 (3.0)	55.5 (3.0)	56.7 (2.1)	56.8 (3.0)	-0.36 [0.45]	-1.24 to 0.52	0.419	56.7 (2.5)	56.9 (2.4)	-0.12 [0.44]	-0.98 to 0.75	0.792
**BVFDT**	30.1 (2.2)	29.7 (1.9)	30.0 (2.7)	29.7 (2.2)	0.05 [0.54]	-1.01 to 1.12	0.923	30.5 (1.7)	30.5 (1.8)	0.12 [0.53]	-0.92 to 1.16	0.818

[a]Corrected for baseline score and age, sex and education in years; [b]Lower is better - negative estimates indicate effects in favor of CT.
Abbreviations: BNT = Boston Naming Test, BVFDT = Benton Visual Form Discrimination Test, COWAT = Controlled Oral Word Association Test, LLT = Location Learning Test, RAVLT = Rey Auditory Verbal Learning Test, RCFT = Rey Complex Figure Test, SCWT = Stroop Color Word Test.

References

1. Scharre DW, Chang SI, Murden RA, et al. Self-administered Gerocognitive Examination (SAGE): a brief cognitive assessment Instrument for mild cognitive impairment (MCI) and early dementia. Alzheimer Dis Assoc Disord 2010; 24(1): 64-71.
2. Kulisevsky J, Fernandez de Bobadilla R, Pagonabarraga J, et al. Measuring functional impact of cognitive impairment: validation of the Parkinson's disease cognitive functional rating scale. Parkinsonism Relat Disord 2013; 19(9): 812-817.
3. Brown RL, Rounds LA. Conjoint screening questionnaires for alcohol and other drug abuse: criterion validity in a primary care practice. Wis Med J 1995; 94(3): 135-140.
4. Ewing JA. Detecting alcoholism. The CAGE questionnaire. JAMA 1984; 252(14): 1905-1907.
5. Nasreddine ZS, Phillips NA, Bedirian V, et al. The Montreal Cognitive Assessment, MoCA: a brief screening tool for mild cognitive impairment. J Am Geriatr Soc 2005; 53(4): 695-699.
6. Voss T, Bahr D, Cummings J, Mills R, Ravina B, Williams H. Performance of a shortened Scale for Assessment of Positive Symptoms for Parkinson's disease psychosis. Parkinsonism Relat Disord 2013; 19(3): 295-299.
7. Beck AT, Ward CH, Mendelson M, Mock J, Erbaugh J. An inventory for measuring depression. Arch Gen Psychiatry 1961; 4: 561-571.
8. Hoehn MM, Yahr MD. Parkinsonism: onset, progression and mortality. Neurology 1967; 17(5): 427-442.
9. Shallice T. Specific impairments of planning. Philos Trans R Soc Lond B Biol Sci 1982; 298(1089): 199-209.
10. Folstein MF, Folstein SE, McHugh PR. "Mini-mental state". A practical method for grading the cognitive state of patients for the clinician. J Psychiatr Res 1975; 12(3): 189-198.
11. Williams-Gray CH, Evans JR, Goris A, et al. The distinct cognitive syndromes of Parkinson's disease: 5 year follow-up of the CamPaIGN cohort. Brain 2009; 132(Pt 11): 2958-2969.
12. Hammes JGW. De Stroop Kleur-Woord Test. Handleiding. Amsterdam: Pearson Assessment and Information B.V., 1971.
13. Schmand B, Groenink S, Van den Dungen M. Letterfluency: psychometrische eigenschappen en Nederlandse normen. Tijdschr Gerontol Geriatr 2008; 39(2): 64-74.
14. Wechsler D. Wechsler adult intelligence scale - third edition. Dutch version. Amsterdam: Pearson Assessment and Information B.V., 2000.
15. Saan RJ, Deelman BG. De 15-woordentest A en B (een voorlopige handleiding). Groningen: Afdeling Neuropsychologie, AZG, 1986.
16. Kessels RP, Nys GM, Brands AM, van Zandvoort MJ. [The Location Learning Test as a measure of spatial memory: applicability of a modified administration procedure and normative data]. Tijdschr Gerontol Geriatr 2004; 35(4): 147-152.
17. Kaplan E, Goodglass H, Weintraub S. Boston naming test: Pro-ed, 2001.
18. Luteijn F, Barelds D. Herziening van de GIT. Handleiding bij de GIT-2. Harcourt Publishers, Amsterdam; 2005.
19. Meyers JE, Meyers KR. Rey Complex Figure Test and recognition trial professional manual: Psychological Assessment Resources, 1995.
20. Benton AL, Sivan AB, deS Hamsher K, Varney NR. Contributions to neuropsychological assessment: A clinical manual: Oxford University Press, USA, 1994.
21. Broadbent DE, Cooper PF, FitzGerald P, Parkes KR. The Cognitive Failures Questionnaire (CFQ) and its correlates. Br J Clin Psychol 1982; 21 (Pt 1): 1-16.
22. Starkstein SE, Mayberg HS, Preziosi TJ, Andrezejewski P, Leiguarda R, Robinson RG. Reliability, validity, and clinical correlates of apathy in Parkinson's disease. J Neuropsychiatry Clin Neurosci 1992; 4(2): 134-139.
23. Leentjens AF, Dujardin K, Pontone GM, Starkstein SE, Weintraub D, Martinez-Martin P. The Parkinson Anxiety Scale (PAS): development and validation of a new anxiety scale. Mov Disord 2014; 29(8): 1035-1043.
24. Weintraub D, Mamikonyan E, Papay K, Shea Ja, Xie SX, Siderowf A. Questionnaire for impulsive-compulsive disorders in Parkinson's Disease-Rating Scale. Mov Disord 2012; 27: 242-247.

25. McLean G, Tobias M. The New Zealand physical activity questionnaires: Report on the validation and use of the NZPAQ-LF and NZPAQ-SF self-report physical activity survey instruments. . New Zealand: SPARC, 2004.
26. Devilly GJ, Borkovec TD. Psychometric properties of the credibility/expectancy questionnaire. J Behav Ther Exp Psychiatry 2000; 31(2): 73-86.
27. Nucci M, Mapelli D, Mondini S. Cognitive Reserve Index questionnaire (CRIq): a new instrument for measuring cognitive reserve. Aging Clin Exp Res 2012; 24(3): 218-226.
28. Fahn S, Elton RL, UPDRS Development Committee A. Unified Parkinson's Disease Rating Scale. In: Fahn S, Marsden CD, Calne DB, Goldstein M, eds. Recent developments in Parkinson's disease. Florham Park, NJ: Macmillian Healthcare Information, 1987:153-163, 293-304.
29. Olde Dubbelink KT, Stoffers D, Deijen JB, Twisk JW, Stam CJ, Berendse HW. Cognitive decline in Parkinson's disease is associated with slowing of resting-state brain activity: a longitudinal study. Neurobiol Aging 2013; 34(2): 408-418.
